# Nephropathic Cystinosis: Pathogenic Roles of Inflammation and Potential for New Therapies

**DOI:** 10.3390/cells11020190

**Published:** 2022-01-06

**Authors:** Mohamed A. Elmonem, Koenraad R. P. Veys, Giusi Prencipe

**Affiliations:** 1 Department of Clinical and Chemical Pathology, Faculty of Medicine, Cairo University, Cairo 11628, Egypt; 2 Egypt Center for Research and Regenerative Medicine (ECRRM), Cairo 11517, Egypt; 3 Laboratory of Pediatric Nephrology, Department of Development & Regeneration, KU Leuven, 3000 Leuven, Belgium; koenraad.veys@uzleuven.be; 4 Department of Pediatrics, AZ Delta Campus, 8820 Torhout, Belgium; 5 Laboratory of Immuno-Rheumatology, Bambino Gesù Children’s Hospital, IRCCS, 00165 Rome, Italy; giusi.prencipe@opbg.net

**Keywords:** macrophages, inflammasome, proximal tubular cells, endocytosis, autophagy, apoptosis, chitotriosidase, interleukins, galectin-3, cysteamine, novel therapies

## Abstract

The activation of several inflammatory pathways has recently been documented in patients and different cellular and animal models of nephropathic cystinosis. Upregulated inflammatory signals interact with many pathogenic aspects of the disease, such as enhanced oxidative stress, abnormal autophagy, inflammatory cell recruitment, enhanced cell death, and tissue fibrosis. Cysteamine, the only approved specific therapy for cystinosis, ameliorates many but not all pathogenic aspects of the disease. In the current review, we summarize the inflammatory mechanisms involved in cystinosis and their potential impact on the disease pathogenesis and progression. We further elaborate on the crosstalk between inflammation, autophagy, and apoptosis, and discuss the potential of experimental drugs for suppressing the inflammatory signals in cystinosis.

## 1. Introduction

Crystallopathies, defined as diseases that involve the accumulation of intrinsic or environmental crystals or microparticles in the pathogenesis of tissue injury, are pathologically multifactorial syndromes that have been linked to the production of reactive oxygen species, immune cell recruitment and activation, and increased expression of various inflammatory cascade molecules [1,2]. The process of crystallization within cells is usually a very slow process, but when crystals form, they elicit direct cytotoxic effects, starting an auto-amplifying inflammatory loop commonly ending in cell death [2]. Crystals are commonly heavily deposited in the kidney, being the main excretory organ for most minerals and organic compounds that can form crystals. These include calcium oxalate, calcium phosphate, anhydrous uric acid, magnesium ammonium phosphate, sodium urate, and cystine. In many cases, crystal deposition is caused by lifestyle factors, such as poor dietary choices and improper hydration [3]. However, some of these disorders are hereditary in nature, including nephropathic cystinosis and primary hyperoxaluria, which are systemic diseases primarily affecting the kidney.

Nephropathic cystinosis is a monogenic autosomal recessive lysosomal storage disorder caused by variants in the *CTNS* gene, which codes for cystinosin—the cystine lysosomal symporter [4]. Defective cystinosin function leads to a multisystemic intra-lysosomal cystine accumulation. The disease commonly affects the kidneys during the first year of life through proximal tubular damage, leading to renal Fanconi syndrome, followed by progressive glomerular damage, and almost invariably ends in kidney failure. This usually requires kidney replacement therapy or kidney transplantation by the end of the first decade of life, if the child is not treated. Other affected organs include the eyes, thyroid, pancreas, gonads, muscles, and central nervous system (CNS), amongst others [5,6]. There are three major phenotypes: the infantile nephropathic phenotype (MIM #219800), which constitutes the majority of cases and appears during infancy and progresses to kidney failure rapidly if left untreated; the juvenile nephropathic phenotype (MIM #219900), which also affects the kidney but at a later age and is more slowly progressive; and the ocular or non-nephropathic phenotype (MIM #219750), which spares the kidney and usually manifests during adulthood [7]. 

Apart from the direct harmful effects of cystine crystal accumulation, several pathogenic processes affect cystinotic cells. Enhanced apoptosis is evident in vivo and in vitro in many cystinotic cell types, such as proximal tubular epithelial cells (PTECs), fibroblasts, and podocytes [8,9]. Both deficient ATP and cAMP levels were detected in cystinotic cells and were associated with mitochondrial dysfunction [10,11,12]. The enhanced motility and defective adhesion of cells leading to the loss of podocytes and PTECs in urine is a pathogenic process that contributes to the rapid progression towards kidney failure [9]. Defective endocytic trafficking and impaired proteolysis are commonly attributable to the evident lysosomal dysfunction in cystinosis [13]. Cystinosin further plays an important role in lysosomal homeostasis involving the mammalian target of the rapamycin complex 1 (mTORC1) and transcription factor EB (TFEB) [14,15]. When cystinosin is defective or missing, lysosomal signaling is disturbed resulting in altered regulation of lysosomal biogenesis and clearance and affecting both autophagy and anabolism [11,15,16]. 

Inflammation has recently been revealed as a major contributing mechanism to the pathogenesis and progression of both the renal and systemic involvement in cystinosis [17,18]. Cystine crystal accumulation, macrophage activation, enhanced oxidative stress, and inflammasome pathway activation are all driving factors behind the vicious cycle of cellular inflammation in cystinosis, which, no matter what the affected organ is, commonly ends in tissue fibrosis and loss of function. In the current review, we will discuss the different aspects of the inflammation process in cystinosis. We will detail the mechanisms behind inflammation, the effects of cysteamine, the current standard therapy for cystinosis on the inflammatory process, and, finally, the concept of targeting the inflammation in cystinosis as a novel potential therapeutic approach for the disease.

## 2. Role of Macrophages in Cystinosis

The kidneys of cystinotic patients and *Ctns*^−/−^mice are characterized by tubular atrophy and interstitial fibrosis, which are focally associated with inflammatory mononuclear infiltrates and largely consist of CD68 positive monocytes/macrophages [19,20,21]. Macrophages, together with dendritic cells, are the most abundant types of leukocytes present in the normal kidney [22]. In addition to playing a pivotal role in the innate immune responses, macrophages are critical mediators for regulating the maintenance of tissue homeostasis [23]. Indeed, they are versatile players in various processes, including balancing the development of fibrosis versus tissue repair and remodeling, clearance of cellular debris, angiogenesis, and various metabolic functions, such as glycolysis and oxidative phosphorylation [24]. In diseased kidneys, macrophages are increased in number and are key players in renal injury, inflammation, and the development of fibrosis [25].

**Figure 1:**
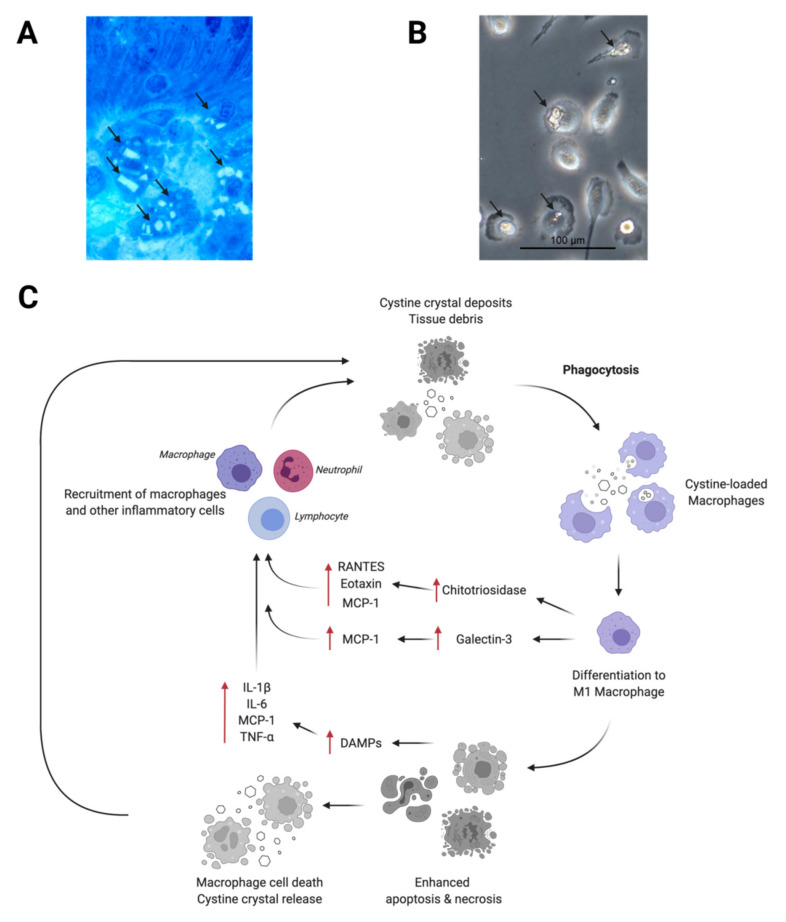
Macrophage activation and tissue injury in cystinosis. (**A**) Tissue cystine crystal accumulation in gastric mucosa (Toludine blue). Hexagonal and rhomboid cystine crystals are visible inside interstitial macrophages (arrows). (**B**) In vitro, incubation of macrophages with cystine crystals is followed by phagocytosis of the crystals (arrows). (**C**) A schematic diagram summarizing the mechanisms of macrophage recruitment and injury in tissues of nephropathic cystinosis patients. DAMPs, damage-associated molecular patterns; IL-1β, interleukin 1β; IL-6, interleukin 6; MCP-1, monocyte chemoattractant protein-1; RANTES, Regulated on Activation, Normal T Expressed and Secreted; TNF-α, tumor necrosis factor-α.

Several bits of direct and indirect evidence strongly suggest a role for macrophages in the pathogenesis of cystinosis. Indeed, in cystinotic patients and in *Ctns*^−/−^ mice, cystine crystals have been observed in the macrophages of most organs, including bone marrow, kidneys, liver, skin, and gastrointestinal mucosa [25,26,27,28] (Figure 1A). In in vitro studies, monocytes/macrophages displayed a great ability to phagocytize cystine crystal deposits [17,18] (Figure 1B), suggesting that, in these cells, cystine crystal accumulation is in part due to the dysfunction or absence of cystinosin, and in part due to the phagocytosis of the dying cells releasing their intracellular contents including cystine crystals. Cystinotic macrophages are unable to dissolve or get rid of the phagocytized cystine crystals because they have the same genetic and biochemical defect as other cytinotic cells, thus they tend to enter a non-ending cycle of cystine crystal deposition, wherein they send signals for the recruitment of other inflammatory cells and finally die [17].

Based on their activation status and functions, macrophages are classified as classically activated macrophages (M1 macrophages), wound-healing macrophages (M2a macrophages), and regulatory macrophages (M2c macrophages) [29]. In *Ctns*^−/−^ mouse kidneys, macrophages mainly display an M1-like pro-inflammatory profile [21]. Accordingly, phagocytosis of cystine crystals has been demonstrated to lead to the activation of monocytes/macrophages. Indeed, the uptake of cystine crystals by cultured human monocytes and mature macrophages induced the production of the pro-inflammatory cytokines, interleukin-1β (IL-1β) [18], and tumor necrosis factor-alpha (TNF-α) [17]. Consistent with the results obtained in vitro, cystinotic patients had significantly elevated plasma levels of IL-1β and an increase in *IL1B* mRNA expression levels in the peripheral blood mononuclear cells (PBMCs) was observed [18]. Moreover, a positive correlation between the intracellular cystine levels in polymorphonuclear leukocytes, at the time of blood sampling, and *IL1B* mRNA expression levels in PBMCs was also found [18]. 

Further supporting a role for cystine crystals as the activators of inflammatory responses in macrophages, the elevated levels of chitotriosidase enzyme activity were also reported in the supernatants of control macrophages incubated with an increasing concentration of cystine crystals recently [17,30]. Accordingly, an elevation in the chitotriosidase enzyme activity was also reported in patients with cystinosis, as well as in *Ctns*^−/−^ mice [17] and in mutant zebrafish larvae (unpublished data). Chitotriosidase is an enzyme involved in the degradation of chitin (chitinase) that is expressed by activated macrophages, of which the activity has been found to be elevated in various lysosomal storage diseases, including Gaucher’s disease, galactosialidosis, Niemann–Pick A/B and C diseases, cholesteryl ester storage disease, and several others [31,32]. Interestingly, chitotriosidase has been proposed as a novel biomarker for therapeutic monitoring and as a predictor of disease severity in nephropathic cystinosis [17]. Indeed, in a longitudinal study involving 61 cystinotic patients treated with cysteamine, the plasma chitotriosidase enzyme activity was significantly correlated with the white blood cells’ cystine levels and with a number of extrarenal complications, suggesting that chitotriosidase enzyme activity, by reflecting the systemic cystine crystal-induced inflammation, gave better guidance on the whole-body cystine burden and, hence, compliance with cystine-depleting therapy [30].

Further supporting the role of macrophage-mediated inflammation in the progression of kidney disease in cystinosis, a recent study demonstrated a novel role for cystinosin, independent of the accumulation of cystine crystals, in regulating the localization and degradation of the β-galactosidase-binding protein family member Galectin-3 [21]. Galectin-3 is an inflammatory mediator that plays a role in acute and chronic inflammation by attracting monocytes/macrophages in diseased tissues. In *Ctns*^−/−^mice, the genetic deletion of *Gal3* led to decreased monocytes/macrophages infiltration in the kidney, amelioration of the renal functions, and to a significant reduction in the circulating levels of the monocyte chemoattractant protein-1 (MCP-1) cytokine. Accordingly, increased serum levels of MCP-1 also have been found in *Ctns*^−/−^ mice and in patients with cystinosis, despite cysteamine treatment [21].

Although the exact nature of the immunostimulatory signals activating macrophages in cystinosis is still unknown, it is well documented that deposits of crystals, including cystine crystals, are able to induce cellular necroptosis, thereby leading to a release of damage-associated molecular patterns (DAMPs) [33]. DAMPs are endogenous danger molecules that initiate inflammatory responses by activating the pattern recognition receptors (PRRs), such as Toll-like and Nod-like receptors (TLRs and NLRs) [34]. Both TLRs and NLRs are expressed in the innate immune cells, such as macrophages and dendritic cells, as well as in non-immune cells, such as epithelial cells and fibroblasts. Therefore, it is reasonable to hypothesize that in cystinosis the cells damaged by the crystals release DAMPs that, in turn, initiate inflammatory responses by triggering various cells, including tissue-resident macrophages, and result in the release of pro-inflammatory cytokines and chemokines—such as IL-1β, IL-6, TNF-α, and MCP-1. The released cytokines and chemokines promote the recruitment of inflammatory cells that contribute to the exacerbation of the tissue injury (Figure 1C).

Altogether, this evidence strongly supports a role for the involvement of activated macrophages in the progression of tissue damage in cystinosis, particularly in the kidney.

## 3. Inflammasome Activation in Cystinosis

One of the most recently identified mechanisms of action, by which crystals induce kidney injury in both acute kidney injury (AKI) and chronic kidney disease (CKD), is the activation of the intracellular sensor inflammasome [35]. Inflammasomes are multiprotein complexes that play a major role in innate immune responses by controlling the caspase-1–dependent proteolytic maturation and release of the pro-inflammatory cytokines IL-1β and IL-18 [36]. 

Inflammasomes sense a wide variety of pathogen-associated molecular patterns as well as of DAMPs, including host-derived crystalline moieties [37,38]. Indeed, endogenous crystals or particles, such as monosodium urate, calcium phosphate, hydroxyapatite, silica, asbestos, cholesterol crystals, and calcium oxalate, have been found to act as agonists of the best characterized inflammasome, NLRP3 [39]. Although the exact mechanism by which NLRP3 is activated by several stimuli has not been fully clarified, NLRP3 activation by crystalline structures has been demonstrated to require lysosomal membrane destabilization, cathepsin B release into the cytosol, the generation of reactive oxygen species (ROS), an increase in cytosolic Ca^2+^, and potassium efflux [40]. Similar to other crystalline inflammasome activators in in vitro experiments, cystine crystals are able to activate the inflammasome system in control of the monocytes and to induce caspase-1-dependent IL-1β secretion through a mechanism involving cathepsin B leakage, ROS production, and potassium efflux [18]. Consistent with this in vitro data in cystinotic patients, the circulating levels of the inflammasome-induced cytokines IL-1β and IL-18 were found to be significantly elevated when compared with those observed in healthy subjects. Further supporting a role for inflammasome activation in the pathogenesis of cystinosis in *Ctns*^−/−^mice, there was a significant increase in the renal expression of inflammasome-related genes, and an increase in the circulating levels of IL-18 was also observed [35]. Recently, the increased mRNA and protein levels of IL-1β were also found in the affected skeletal muscles of *Ctns*^−/−^ mice [41]. However, to date, it remains to be studied in which cells, in vivo, in the cystinotic kidney and other affected organs, the inflammasome is activated. Several studies demonstrated that, in addition to the renal resident immune cells, PTECs, endothelial, and mesangial cells also express TLRs and NLRs and display the ability for releasing inflammatory molecules, including the IL-1β cytokine [42]. In this context, inflammasome-independent roles for NLRPs in the progression of chronic kidney diseases have also been reported [43]. Indeed, in addition to activating inflammasomes, NLRPs are also involved in the modulation of the inflammatory pathways in immune and non-immune cells, wherein they regulate the activity of the transcription factor NF-κB [44]. Recently, markedly elevated levels of the NOD-like receptor family member NLRP2 have been found in PTECs, both in primary cell cultures and in kidney biopsies, from cystinotic patients [45]. In particular, in vitro studies on cystinotic PTEC revealed a role for NLRP2 in amplifying the pro-inflammatory and profibrotic responses through the modulation of NF-kB activity, thereby unveiling an additional potential mechanism involving inflammation in the pathogenesis of cystinosis.

**Figure 2:**
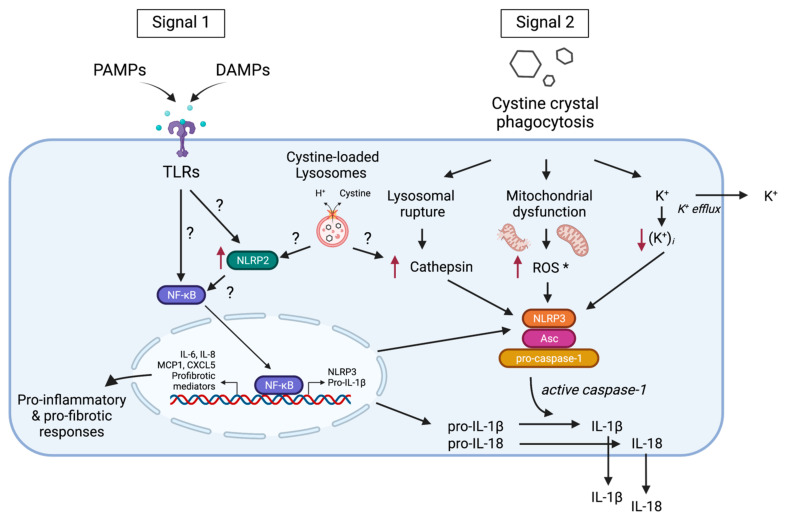
Activation of inflammasome-dependent and independent mechanisms in cystinosis. In vitro, in cultured monocytes, cystine crystals act as an NLRP3 agonist. Inflammasome activation requires two signals. Signal 1 is triggered by PAMPs or DAMPs through TLR/NLR activation and leads to NF-κB-dependent transcriptional activation of inflammasome components, including NLRP3, and IL-1β.Signal 2 is triggered by cystine crystals phagocytosis, causing K^+^ efflux leading to hypokalemia, oxidative stress activation by ROS, or lysosomal rupture, and leads to the activation of the NLRP3 inflammasome, which ultimately results in the cleavage and secretion of the pro-inflammatory cytokines IL-1β and IL-18. A role for the cystine-loaded lysosome in activating inflammasomes has not been demonstrated. Inflammasome-independent mechanisms have also been described in human cystinotic PTECs, in which markedly high levels of NLRP2 have been observed. NLRP2 amplifies pro-inflammatory and profibrotic responses through activation of the NF-κB signaling pathway. CXCL5, C-X-C motif chemokine 5; DAMPs, damage-associated molecular patterns; IL, interleukin; MCP-1, monocyte chemoattractant protein-1; NF-κB, nuclear factor kappa light chain enhancerof activated B cells; NLRP3, NLR family pyrin domain containing 3; PAMPs, pathogen-associated molecular patterns; ROS, reactive oxygen species; TLRs, Toll-like receptors.

Altogether, these studies strongly support a role for inflammasome/NLRs-mediated inflammation in contributing to the pathogenesis and/or progression of cystinosis (Figure 2), and, therefore, provide the rationale for potential novel targets in the treatment of cystinosis.

## 4. Interplay between Inflammation, Autophagy and Apoptosis in Cystinosis

Inflammatory cytokines, such as TNF-α and IL-1β, have long been associated with the stimulation of programmed cell death, or apoptosis, in various diseases [46,47,48], although the mechanisms for cell death in response to inflammatory cytokines are thought to be somehow distinct from those of genuine apoptosis [49]. Altered autophagy signals have recently been implicated in the crosstalk between inflammatory signals and apoptotic signals [50]. Autophagy is an important physiological cellular process involved in the restoration of energy homeostasis through the catabolism and recycling of dysfunctional or unneeded proteins and cellular organelles to improve cell survival upon exposure to various stress conditions. Furthermore, it may eliminate the specific stress triggered by damaged organelles, such as the endoplasmic reticulum (ER) or mitochondria [51,52]. Essentially, whether the cells survive or die during inflammation is largely dependent on the intricate balance between the pro-survival mechanisms of autophagy, when functioning properly, and the pro-death mechanisms of apoptosis or necroptosis [50]. 

In cystinosis, structurally and functionally abnormal mitochondria are an especially important aspect of the disease. As a major source of ROS, mitochondria are particularly susceptible to oxidative stress resulting from the inflammatory processes triggered by cystine accumulation and crystallization. This usually leads to the abnormal induction of mitochondrial autophagy (mitophagy), particularly in cystinotic PTECs and fibroblasts, with the enhanced expression of the microtubule-associated protein 1 light chain 3 (LC3-II/LC3-I) and beclin-1 [53]. This process was further associated with reduced mitochondrial ATP generation, cell starvation, increased generation of ROS, autophagic flux blockade, and enhanced apoptosis [53,54]. Moreover, mitochondrial dysfunction is usually associated with ER stress, which is a common pathogenic process that is linked to altered autophagy in various lysosomal storage disorders, including cystinosis [55,56]. Furthermore, caspase-4, the apoptotic cascade molecule, was stimulated by ER stress and was significantly enhanced in cystinotic PTECs [57].

Several inflammatory cytokines, such as IL-1β, TNF-α, and INF-γ, can enhance the process of autophagy through the activation of the mammalian target for rapamycin (mTOR) and AMP-activated protein kinase (AMPK) pathways [58]. Moreover, the important autophagy substrate, p62/SQSTM1, has been linked to the stimulation of chronic inflammation in the skin and myometrium through the activation of the NF-κB signaling pathway [59,60]. This molecule has been demonstrated as significantly elevated in the cellular models of cystinosis, particularly in PTECs [61,62,63].

**Figure 3:**
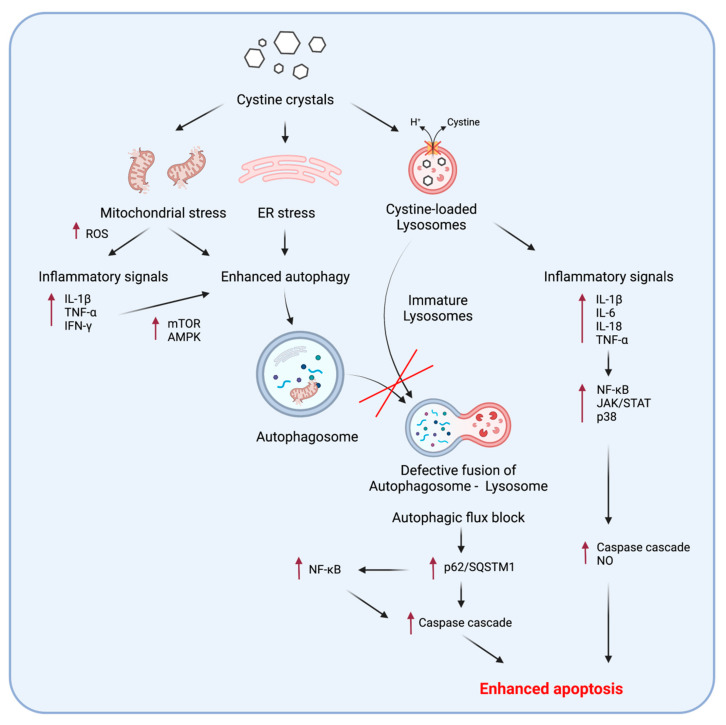
The interplay of inflammation, autophagy, and apoptosis in nephropathic cystinosis cells.A schematic representation of the effects of cystine crystals leading to the disturbance of inflammatory, autophagy, and apoptotic signals, and the disturbance of the crosstalk between these pathways. AMPK, AMP-activated protein kinase; INF-γ, interferon-γ; IL, interleukin; JAK/STAT, Januskinase/signaltransducerandactivatoroftranscription; mTOR, mammalian target of rapamycin;NF-κB, nuclear factor kappa light chain enhancerof activated B cells; NO, nitric oxide; p38, p38 mitogen activated protein kinase; p62/SQSTM1, Sequestosome-1; ROS, reactive oxygen species; TNF-α, tumor necrosis factor-α.

Apoptotic signals can be initiated by both inflammatory cytokines and the autophagy substrate p62/SQSTM1. While p62/SQSTM1 can directly activate the extrinsic caspase pathway through the activation of caspase-8 and followed by caspase-3 [64,65], the inflammatory cytokines, such as IL-1β, IL-18, and TNF-α, stimulate apoptosis mainly by involving downstream signals, such as NF-κB, JAK/STAT, and p38/MAPK pathways, which may directly activate the caspase cascades or apoptosis induced by nitric oxide (NO) [46,66]. Figure 3 summarizes the important points of the crosstalk of inflammation, autophagy, and apoptosis in cystinotic cells.

In conclusion, inflammation in cystinosis disturbs the balance of the autophagy machinery, as both the inhibition of autophagy leading to the incomplete processing of autophagolysosomes and the abnormal induction of autophagy of certain organelles, such as mitochondria and the ER, can eventually lead to enhanced apoptosis. 

## 5. Response to Cysteamine Therapy in Cystinosis

The aminothiol cysteamine (beta-mercaptoethylamine) is currently the only approved specific therapy for nephropathic cystinosis. It was first applied for the treatment of cystinosis in 1976 and was approved by the FDA in 1994. It enters the lysosome and biochemically depletes the accumulated cystine very efficiently through its conversion into cysteine and cysteamine-cysteine–mixed disulfide, which can both exit the lysosome via different transporter mechanisms other than cystinosin, thus overcoming the main genetic and biochemical defects of the disease [4,67,68]. When initiated early in life at a proper dosage, cysteamine can improve the overall prognosis through delaying the development of end stage kidney disease and reducing the incidence of the major extra-renal complications, including hypothyroidism, myopathy, diabetes mellitus, neurological manifestations, and growth retardation. However, while it merely delays progressive kidney dysfunction, it cannot cure renal Fanconi syndrome, nor can it cure any other extra-renal complications when the drug is initiated late, during the course of the disease [69,70].

**Figure 4:**
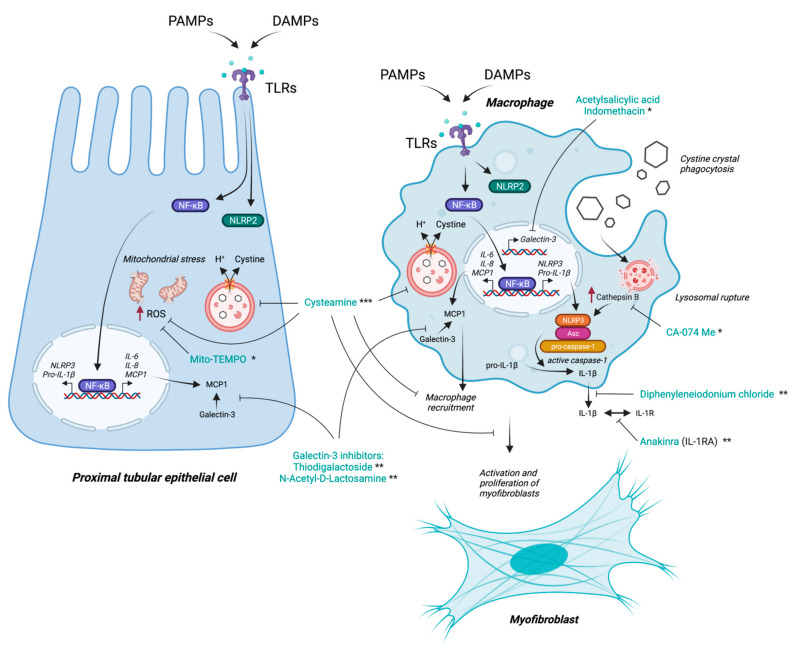
Inflammatory pathways in key cells involved in the pathophysiology of cystinosis and mechanisms of action of current and potential future therapeutic agents. Proximal tubular epithelial cells (PTECs), macrophages, and myofibroblasts are key cell players in the proximal tubular dysfunction and progressive chronic kidney disease, which characterizes cystinosis. Therapeutic agents are depicted with their specific mechanisms of action and classified according to the current evidence. * In vitro cellular models, **: in vivo animal models, ***: in vivo clinical-grade patient data. DAMPs, Damage-associated molecular patterns; IL, interleukin; MCP-1, monocytechemoattractant protein-1; NF-κB, nuclear factor kappa light chain enhancerof activated B cells; NLRP2, NLR family pyrin domain containing 2; PAMPs, pathogen-associated molecular patterns; ROS, reactive oxygen species; TLRs, Toll-like receptors.

Apart from its cystine-lowering effects, which definitely have an enormous impact on decreasing the cellular inflammation, being one of its most powerful driving factors, cysteamine is also a potent antioxidant molecule that can suppress many of the inflammatory cascades following oxidative stress. It can reduce ROS generation, attenuates macrophage recruitment and activity, blocks myofibroblast proliferation and activation, and significantly decreases tissue fibrosis [71] (Figure 4). Cysteamine in low concentrations aids in the transport of the amino acid, cysteine, inside cells, which serves as replenishment machinery for the reduced glutathione—one of the most important intracellular antioxidants [72,73]. In PTECs, cysteamine was able to normalize both cystine levels and the glutathione redox status; however, it could not improve the mitochondrial ATP production or the decreased sodium-dependent phosphate uptake [74]. 

Interestingly, Okamura et al. demonstrated that, in two mouse models of CKD unrelated to cystinosis, cysteamine exerted its reno-protective effects by modulating oxidative stress [71]. Indeed, when macrophages were co-cultured in vitro with apoptotic renal tubular cells, intracellular ROS generation was reduced by 43–52% in the cysteamine-treated macrophages. In addition, cysteamine treatment attenuated the macrophage accumulation in the kidneys, inhibited myofibroblast differentiation and proliferation, and led to significant amelioration of renal fibrosis [71]. Therefore, although data regarding the effects of cysteamine on the inflammatory status of cystinotic patients and of *Ctns*^−/−^ mice are lacking, it is conceivable that in cystinosis the efficacy of cysteamine may also be due, in addition to the lowering of intracellular levels of cystine, to an alternative anti-inflammatory mechanism of action. Cysteamine is further a potent inhibitor of apoptosis in cystinotic cells. This is mainly attributable to its cystine lowering effects [8]; however, its antioxidant and anti-inflammatory properties must play a role in this as well. These beneficial effects are certainly linked to the better preservation of kidney function and the delay of most complications in cystinosis patients on cysteamine therapy.

In contrast, cysteamine treatment had no significant effect on the lysosomal size and distribution or on the altered ultrastructure of the endosomal/lysosomal compartments in cystinosin-deficient cells, but it could partially improve the delayed lysosomal cargo processing in human PTECs [13]. Upon studying the effects of cysteamine therapy on the impaired proximal tubular endocytosis in cystinotic zebrafish, it could significantly increase the amount of low molecular weight dextran reabsorbed in the proximal tubules compared to the untreated group, which is in support of the human cell findings. However, defective megalin expression in cystinotic zebrafish was not restored after cysteamine treatment. Furthermore, both EEA1 (a tethering protein of early endosomes) and the small GTPase Rab11 associated with recycling endosomal trafficking to the plasma membrane were significantly less expressed in the proximal tubules of the cystinosis zebrafish larvae compared to the wild type. Cysteamine treatment did not restore the protein expression of either of them, denoting that cysteamine has a minimal effect on the defective endocytic machinery in cystinosis, which can partially explain its failure in preventing renal Fanconi syndrome [75,76]. Consistent with these observations, the treatment with cysteamine did not normalize the circulating MCP-1 levels that were found significantly increased in the sera of patients with cystinosis compared with healthy donors, which strongly suggests that the absence of cystinosin, rather than cystine accumulation, is responsible for MCP-1 increase in serum [21].

In short, cysteamine is a very beneficial drug for cystinosis patients. It significantly prolongs and improves the quality of their lives; however, it is far from a perfect drug or a cure for the disease. It is true that the majority of its beneficial effects are driven by its cystine-depleting capabilities, but its functional impact on the proximal tubular cells is limited, and its effects on the inflammation/autophagy/apoptosis axis signals in cystinotic cells are not yet fully clarified.

## 6. Targeting Inflammation as a Potential Therapeutic Option in Cystinosis

With the recent perception that inflammatory processes are largely involved in many of the pathogenic aspects of cystinosis, and that inflammation is not only involved in facilitating the progression of the disease through the development of tissue fibrosis and damage, but also in contributing to the disruption of certain cellular functions—such as endocytosis in PTECs—it was logical to assume that targeting the inflammatory signals could be a novel potential therapeutic strategy complementing the conventional cystine-depleting therapies used in cystinosis.

Historically, indomethacin, the non-steroidal anti-inflammatory drug, has been proposed in the management of cystinosis patients. The drug caused clinical improvement by reducing polyuria and polydipsia, improved their general wellbeing, and partially normalized the electrolyte imbalance associated with the disease [77,78]. These beneficial effects are due to the inhibition of prostaglandin synthesis in the renal parenchyma and its partial restoration of the medullary blood flow, thereby enhancing salt reabsorption in the loop of Henle and the collecting tubules, and thus, partially compensating for the proximal tubular losses [78]. Although the studies reporting the use of indomethacin in cystinotic patients are scarce, several centers, particularly in Europe, widely use it in young cystinotic children to improve their renal Fanconi phenotype [78]. In a large multi-centre cohort of 453 cystinosis patients born between 1964 and 2016, the use of indomethacin was not associated with improved kidney function, and it cannot be excluded, though its direct anti-inflammatory effects were diluted in this large cohort, as the dose and the duration of treatment were not specified in this study [79]. In this regard, it is currently recommended to limit indomethacin to the first years of life, when renal water and salt losses are more severe, in order to avoid its potential long-term nephrotoxicity. To the best of our knowledge, the anti-inflammatory effects of indomethacin have never been evaluated at the cellular level either in in vitro or in vivo models of the disease. 

Other, more recent studies have investigated the effects of other potential therapeutic agents on the inflammatory/oxidative stress axis, with the aim to alleviate some of the pathogenic processes of cystinosis in various cellular and animal models. Festa et al. incubated *Ctns^−/−^* PTECs with the mitochondria-localized-oxygen scavenger Mito-TEMPO. The blockage of the mitochondrial ROS/Gα12/Src signaling by this pharmacologic agent prevented the abnormal phosphorylation of ZO-1 and its lysosomal accumulation, which increased its abundance at cell boundaries and restored the differentiation and endocytic uptake of albumin in cystinotic cells [58]. Prencipe et al. targeted the activated NLRP3 inflammasome pathway in cystinosis through the cathepsin B inhibitors CA-074Me and diphenyleneiodonium chloride, which decreased the secretion of IL-1β induced by the cystine crystals in the peripheral blood monocytes and suppressing the inflammatory cascade that should follow, thereby paving the way for another mechanism that can be targeted in cystinosis patients [18]. Cheung et al. studied *Ctns*^−/−^ mice for both the genetic deletion of *Il1b* as well as treatment with the recombinant IL-1 receptor antagonist (IL-1ra) anakinra, which led to an attenuation of the cachexia phenotype in cystinotic mice, supporting the efficacy of the IL-1 anti-inflammatory-targeted therapy on adipose browning and muscle wasting in nephropathic cystinosis [41]. Adding to this effect, Gonzalez et al. demonstrated significant beneficial actions of the leptin receptor blockade, which is a well-known immunomodulatory cytokine and hormone on the cachexia phenotype in *Ctns^−/−^* mice. Inhibiting leptin action in these mice normalized their food intake and weight gain, increased fat and lean mass, decreased metabolic rate, and stabilized energy homeostasis in the adipose tissue and muscle [80]. However, further studies evaluating the efficacy of these drugs, alone and in combination with the available conventional therapies, in ameliorating kidney and other organ damage in cystinosis are needed.

Lobry et al. took advantage of cystinosin involvement in the regulation of the inflammatory mediator galectin-3. Galectin-3 inhibitors, such as thiodigalactoside and N-Acetyl-D-Lactosamine, decreased the interaction of Galectin-3 with MCP-1 in *Ctns^–/–^* mice and successfully limited macrophage recruitment and activation in mouse tissues [21]. Interestingly, non-steroidal anti-inflammatory drugs, such as aspirin and indomethacin, have been found to inhibit galectin-3 expression in human macrophages by inhibiting its transcription [81], which may be partially responsible for the beneficial effects seen with indomethacin in cystinosis patients. 

In summary, the search for therapeutic agents that can interrupt the impact of the inflammation in cystinosis and restore, at least partially, the functionality of important cell types—such as PTECs—is currently a prime focus for cystinosis research. Although cysteamine has significant antioxidant and anti-inflammatory properties, they are clearly not enough in this regard. A complementary therapy, or therapies, that can efficiently suppress the inflammatory process in cystinosis still need to be validated in human individuals. Figure 4 summarizes the main targets of the potential new therapies aiming at suppressing the inflammatory signals in cystinosis.

## 7. Future Perspectives

Nephropathic cystinosis is a complex systemic disease with a lot of layers to its pathogenesis, risk assessment, and management strategy, and thus usually requires a multidisciplinary approach to properly care for the patient. Inflammation is certainly one of the pathogenic layers that has been documented repeatedly in different cellular and animal models of the disease, and in patients with cystinosis as well. However, this involvement in pathogenesis hasn’t yet been translated to a validated aspect of the disease therapeutic strategy. This could be partially due to the recent discovery of the importance of inflammatory signals in cystinotic cell pathology, but it is also due to the fear of the potential kidney damaging effects of known anti-inflammatory agents, particularly when used for long periods in a disease that is originally harming the kidney. NSAIDs, for example, are famous for their counter vasodilatation effect through the inhibition of the cyclooxygenase enzyme, leading to a hemodynamically mediated acute and chronic kidney injury [82,83]. Corticosteroids, on the other hand, although already used for the treatment of many inflammatory kidney disorders, such as IgA nephropathy, lupus nephritis, and interstitial nephritis [84,85,86], are known for their long-term and severe side-effects, which impact the kidneys—among other organs—and may hinder their consideration as a lifelong treatment, even in low doses, for nephropathic cystinosis. To this date, no prospective therapeutic clinical trial has been conducted to address the inflammatory aspect of cystinosis in human patients. 

Novel and experimental anti-inflammatory agents that do not target the cyclooxygenase/prostaglandins axis may provide a good alternative for classic NSAIDs in this regard, such as anakinra and N-Acetyl-D-Lactosamine, which block the biological activity of IL-1 and the binding of galectin-3 with MCP-1, respectively. Moreover, synthetic inhibitors of human chitinase enzymes, including chitotriosidase inhibitors, are currently being evaluated as potential therapeutic modalities in chronic inflammatory conditions, such as interstitial pulmonary fibrosis and inflammatory bowel disease [87,88]. Chitotriosidase plays an important role in abrogating the inflammatory loop in cystinotic tissue, especially through the recruitment and activation of multiple inflammatory cells [17,30,32]. The other active chitinase in human tissue is acidic mammalian chitinase (AMCase), which has also been implicated in the pathogenesis of bronchial asthma and other inflammatory conditions [89]; however, it has never been studied in cystinosis. The recent development of selective chitotriosidase and dual chitinase inhibitors with very good safety profiles [87,90] may provide further hope to address inflammatory disorders in a pathway that may not cause harm to the kidneys. It would be interesting to see the actions of such inhibitors in inflammation-associated conditions primarily affecting the kidney, such as cystinosis.

## 8. Conclusions

Altogether, inflammation is an important aspect of the pathogenesis of cystinosis that has been overlooked for a long time. Cysteamine is an excellent drug that can deplete cystine from the lysosomes and has some antioxidant and anti-inflammatory properties but cannot neutralize the inflammatory cascade in cystinotic cells and cannot restore some basic cellular functions in the kidney proximal tubules. It is time to address the enhanced inflammation in cystinosis with more specific and targeted therapies that can suppress the abnormal inflammatory signals. This may help to interrupt the cystine-induced cell damage/inflammatory cell recruitment cycle and correct, at least partially, some of the cellular abnormalities responsible for renal Fanconi syndrome and kidney failure down the road. It is definitely worth a try.

## Data Availability

No new data were created or analyzed in this study. Data sharing is not applicable to this article.
